# Effects of forest fragmentation on the effective and realized gene flow of Neotropical tree species: implications for genetic conservation

**DOI:** 10.1186/1753-6561-5-S7-O6

**Published:** 2011-09-13

**Authors:** Alexandre Sebbenn

**Affiliations:** 1 Instituto Florestal de São Paulo, Brazil

## 

Tree species are key organisms of forest ecosystems, due their size and extended life, providing the environmental setting for many other living organisms. The levels of genetic diversity and the effective population sizes are essential issues for the maintenance and survival of tree species populations, because they direct adaptation to the current environment and future environmental changes. Forest fragmentation around the world is a fact, affecting many animal and plant populations in all continents. In Brazil, forest fragmentation and selective logging are the two main problems affecting populations of tropical tree species. For example, the Atlantic Forest was intensively destroyed and fragmented during the last century. Today, only between 11 and 17% of the original areas remain, generally split into small forest fragments or isolated trees in pastures and agricultural lands. In the Atlantic Forest, only 3% of the original area of the Araucaria Forest biome in southern Brazil, remains. Forest fragmentation is especially drastic in tropical forest biomes, due to the very high species diversity, associated to low population density (<1 tree/ha). Thus, after forest fragmentation takes place, the remaining fragmented forest may contain the same species diversity, but the populations are strongly reduced, and, in some cases, less than a dozen reproductive individuals remain. The bottleneck (size reduction of the reproductive population) caused by forest fragmentation associated to spatial isolation of the remaining stands may cause the loss of genetic diversity, an increase in inbreeding and relatedness, reducing the effective population size and blocking seeds and pollen migration inside the stands. These hypotheses have been tested in some tropical tree species of the Araucaria Forest and semicidual Atlantic Forest, including *Araucaria angustifolia*, *Copaifera langsdorffii*, *Myracrodruon urundeuva* and *Hymenaea stigonocarpa*, using microsatellite markers and parentage analysis. More specifically, in these studies we tried to answer the following questions: Can spatial isolation of tree populations following forest fragmentation block seed and pollen migration (gene flow)? What is the distance and patterns of seed and pollen dispersal in populations of tree species living within fragmented stands? Is there intra-population spatial genetic structure (SGS) in the adults and regeneration, and where is it higher, in adults or regeneration after forest fragmentation? Can forest fragmentation really reduce the genetic diversity and effective population size and increase the inbreeding and relatedness within populations? What is the necessary number of seed trees to collect seeds aiming at *ex situ* conservation and environmental reforestations plans with a reference effective population size that provides a minimum evolutionary potential?

Knowledge of these issues is crucial to delineate strategies for *in situ* and *ex situ* conservations, tree breeding and environmental restoration plans. As many tropical trees are long lived individuals, established before forest fragmentation had occurred (<100 years), seeds, seedlings and juveniles, established after forest fragmentation have been included in the samples. Thus, these studies have been based on the sampling of all reproductive trees regenerates (realized seed and pollen dispersal), as well as open-pollinated seed (effective pollen dispersal). All adult trees and regenerates were also mapped (x and y coordinates), the diameter at breast height (dbh) and/or total height measured and in the case of dioecious species, all reproductive trees were also sexed. We used a set of microsatellite loci to arrive to a high exclusion power, to guarantee that each candidate parent displays a unique multilocus genotype for parentage analysis. Our results showed an essentially perfect isolation of seed gene flow, with total absence of seed immigration, probably due to the large distance separating the remaining forest fragments, associated to an intensive use of surrounding land (agriculture, highways, urban areas, etc). Within populations, although long distance seed dispersal events were detected, seeds generally disperse intensively near the mother trees following a classic isolation by distance model. These hypotheses have also been supported by studies of SGS in both adults and juveniles and mating system, revealing significant rates of mating among relatives. In contrast, substantial realized and effective pollen immigrations have been detected in the studied species (ranging from 4 to 10%), evidencing longer-distance pollen dispersal than seed dispersal. Within populations, although the estimates are downward biased due to a substantial rate of pollen immigration in the stands, pollen generally disperses with high intensity at short distances, in a near neighbour pollen dispersal pattern, determined by the individual flowering patterns and behaviour of pollination vectors. Matings are not random, due to selfing, mating among relatives and correlated mating, resulting in higher inbreeding relatedness and lower effective population size in open-pollinated seeds than expected under a panmitic model. High inbreeding and relatedness and lower effective population size were also seen in the regeneration. Lower genetic diversity has been found in seeds, seedlings and juveniles when compared to the adult population, suggesting the occurrence of genetic drift. Taken together our results in the targeted species has indicated a requirement to maintain the genetic connectivity among the forest fragments using strategies of reforestation in riparian areas and forest corridors. Our results also suggest the need to collect seeds from a large number of trees for ex situ conservation, tree breeding and environmental reforestation, due to a typically limited effective population size contained in open-pollinated individuals. This small number of species studied so far does not represent all the genetic, demographic, reproductive and ecological characteristics of the tree diversity in the Atlantic Forest. To provide a stronger support to our hypotheses and results to date, new studies of gene flow, mating system, SGS, genetic diversity and effective population size are underway with other species. Finally, it is never enough to reiterate the importance of conserving tropical tree diversity for future generations so that they can benefit from them as we currently do. Although, *in situ* conservation is the indicated method for this purpose, conserving not only the target species, but also other organism in the same biome using *ex situ* strategies is also necessary due the continued fragmentation and degradation of the Atlantic Forest.

